# Domestic Violence on the Italian Screen: Representation and Resistance

**DOI:** 10.1177/10778012231162036

**Published:** 2023-03-14

**Authors:** Bernadette Luciano

**Affiliations:** 1 School of Cultures, Languages and Linguistics, 1415 University of Auckland, Auckland, New Zealand

**Keywords:** contemporary Italian cinema, domestic violence, female solidarity in film

## Abstract

This article looks at how contemporary Italian films address issues of the tragedy and horror of domestic abuse and attempt to create a space for reflection and change. Ferzan Ozpetek's *(*2008*) Un giorno perfetto (A Perfect Day)*, Andrès Arce Maldonado's *(*2017*) Dentro (Inside), and* Ivano De Matteo’s (2016) *La vita possibile* (*A Possible Life*) ultimately challenge the perception of domestic violence as a fixed and unchangeable condition for women. The films explore how female solidarity, empathy, and care can serve as pathways to combatting the isolation, guilt, and associated emotions that prevent women from seeking or finding *la vita possibile*.

As numerous films and docudramas dealing with domestic violence have proliferated on international screens over recent decades, scholarly works have also begun to consider how images and narratives of such violence reflect social dynamics of power and subordination. In 2014, Silvia Lelli and Matilde Gagliardo began a long-term documentary project entitled *Violenza invisibile*, which aimed to explore the many forms of “less visible” violence against women in Italy, violence embedded and indeed normalized in everyday cultural practices. The documentary was motivated at least partially in response to often misrepresented or sensationalized accounts of domestic violence by the media and offers instead a more analytical and multi-dimensional examination of domestic violence and its implications. The interviews for the project enabled women who had broken away from violent relationships and men who had abused women to talk about their experiences, thus creating a space to address what the filmmakers described as a previously invisible social tragedy. The film was made with an informative and socio-educational agenda that broke down taboos about the open discussion of domestic violence and became a catalyst for public debates and forums. The end goal of the project was to contribute to a widespread understanding of the personal and social dimensions of domestic violence and to be empowering for women.

In this article, I examine how mainstream Italian fiction films, which attract wider audiences through director and actor name recognition and greater cinematic distribution and online streaming venues, might contribute to a similar social agenda. The three films central to this article highlight what national statistics reveal: the pervasive nature of a national tragedy, drawing attention to domestic violence as symptomatic of a culture where violence against women in the domestic space remains invisible because it is still acceptable, because male authority is still sanctioned, and women all too often accept living with such violence for numerous and complex reasons ranging from social expectations to material or psychological dependence.^1^ Ferzan Ozpetek's (2008) *Un giorno perfetto* (*A Perfect Day*), Andrès Arce Maldonado's (2017) *Dentro* (*Inside*), and Ivano De Matteo's (2016) *La vita possibile* (*A Possible Life*), while generically and stylistically distinct from each other, provide different perspectives on domestic violence and expose its pervasive and transversal nature that cuts across age, ethnicity, and class. At the same time, in the unravelling of their narratives, the filmmakers ultimately challenge the conception that domestic violence is a fixed and an unchangeable condition for women*,* providing instead empowering while not overly optimistic conclusions*.* In these films, the protagonists make the risky and dangerous choice of leaving violent partners, mobilized by the agency they achieve through various forms of female solidarity that “enable vulnerable and violated bodies [to] be reconfigured and liberated from the defensive history to which they have been consigned” (Marsden, 2004, p. 308).

## Domestic Violence in Mainstream Italian Cinema

Domestic violence has been represented in either main or peripheral storylines in the history of Italian cinema in ways that reflect prevailing attitudes about sexual roles and sexual relationships. In pre-feminist Italian cinema, we find the omnipresent threat of violence against women and, in particular, of intimate partner violence as a reflection of a highly patriarchal society and indeed as an “excess” of normal male behavior. As Phyllis Frus (2001) argues in her chapter on domestic violence in American film, battering “is not deviant behavior; it is merely excessive—an intensification of the system that gives men control of their households” (p. 229). In Italian cinema, the camera's gaze on women has both explicitly and obliquely represented, perpetuated, and to some extent condoned the violence women are continually subjected to (or risk being subjected to) in both the public and private space. If on the one hand a number of films by male directors in the post-war period turned their attention to the women of that era and to their increased access to the public space, those same women's ability to inhabit and traverse that space unhindered was hampered as they were subjected to the normalized and acceptable behavior of the predatory male. As John Rhodes (2017) notes, Alberto Lattuada's short film *Gli italiani si voltano* (*The Italians Turn Around*), the final episode of the 1953 collective film *Love In the City*, voyeuristically tracks the lives of a number of mobile women protagonists during the course of a single day as they move (walking or on buses) around the city of Rome. The film begins with a series of shots of women opening windows, exiting doors, and stepping out from behind the private into the public space. The camera not only gazes at these women's bodies or parts of their bodies, but seemingly stalks them as they navigate the city of Rome; according to Rhodes, “the film is nearly pornographic if not perverse in its obsessive interest in these women's bodies” (p. 413). As males enter the space of the film, the camera shows how these women are preyed upon not only by the camera but diegetically by male characters who cannot seem to control themselves; they turn and stare at the women and in some cases end up pursuing them. In fact, as Kaplan (1983) notes in her study on the male gaze in cinema: “[M]en do not simply look; their gaze carries with it the power of action and of possession that is lacking in the female gaze. Women receive and return a gaze, but cannot act on it” (p. 32).

The particularly disturbing episode that ends the short film features a young woman who boards a crowded bus in front of the Trastevere train station. The camera follows her as she attempts to free herself from the gaze and the physical groping of a bespectacled middle-aged man who follows her onto the bus. The bus slowly empties out as it approaches the end of the line where both disembark in a deserted square. The mood becomes increasingly eerie as the woman hurries to her apartment building and manages to get safely inside at which point the man stops, stares at the building, and walks away. This episode emphasizes the predatory nature of both the camera and the male protagonists who own the public space and are allowed to hunt and haunt the women who are trying to find their space within it. As Rhodes emphasizes in his analysis, the film exhibits women's “vulnerability to men in urban space (as well as their ingenuity in defending themselves against men in urban space)” (p. 420).

While films in the 1960s and 1970s increasingly drew attention to changing gender roles and relations,^2^ a number of Italian comedies of the period position women as remaining vulnerable inside the private space. This is represented by both the physical interior of the home and by the institution of marriage where any behavior exhibited by a man that ensures his authority over his wife is normalized and justified. The normalization of such behavior may be in part due to what Niahm Cullen (2019) defines as the jealousy pandemic in the years of the Italian economic miracle and into the 1970s when films and magazines were proliferating popular love stories that frequently featured jealousy as a narrative device (p. 132).^3^ At the root of this jealousy was not so much passion, but the anxiety and uncertainty caused by changes in traditional gender roles, which allowed women more freedom and threatened male control. It was not unusual for this obsession for control to lead to physical or psychological abuse (p. 158).

One example of the representation of abusive behavior linked to male obsession for control and experienced by women within the interior spaces of home and marriage is Mario Monicelli's (1974) film *Romanzo popolare* (*Come Home and Meet My Wife*). In the film, the acclaimed actor, Ugo Tognazzi, plays the role of a husband who claims to be a “liberated man” with a 1970s mentality who encourages his liberated wife Vincenzina, played by Ornella Muti, to reveal the details of her trysts with her lover. The more Vincenzina confesses, the more the husband descends into an uncontrollable state of jealousy until he finally pauses, lights a cigarette, and explodes into a fit of rage. The abuse brought to the screen begins with a series of verbal insults that dehumanize her and evolve into physical threats. Like a hunter stalking his prey, he crawls after her on the floor, threatening to strangle her until she finally manages to run away from him into the bedroom where she grabs the screaming baby and escapes. In a similar vein, in *Amore mio aiutami* (*Help Me, My Love*), a 1969 film comedy directed by Alberto Sordi, a jealous husband played by Sordi himself, drags his wife Raffaella, played by Monica Vitti, onto the sand dunes of Sabaudia and demands that she repeat she is in love with her lover. With each admission, he strikes her causing her to bleed. At the time, such representations of domestic violence were not only deemed to be acceptable cinematic material, but were meant to elicit laughter from the audience due at least in part to the fact that the films’ protagonists were played by Italian actors Ugo Tognazzi and Alberto Sordi, who represented the stereotypical middle-class Italian male, a sort of anti-hero whose displays of Italian masculinity oscillate between inept and monstrous behavior. While the female characters played a role in the revelation of this ineptness (Landy, 2020, p. 168), it was the male stars who performed roles that contributed to “that […] panoply of egotistical, criminal, vain and cheating males” (Bayman, 2017, p. 192). As a result, in the comedies cited above as in other films of the time, wife beating is not represented as operating outside acceptable boundaries, but is condoned by the masculine culture codes of the time whereby domestic violence serves to ensure the success of the male's attempt to contain his wife, regain authority, and restore social order. Indeed, in one extreme case, violence is actually portrayed as beneficial to the female character. As Daniela Cavallaro (2008) notes, in Nanny Loy's 1959 film *Audace colpo dei soliti ignoti* (*Hold-up à la Milanaise*), when the slapped female regains consciousness after hitting her head on a suitcase, she has miraculously lost her speech defect, suggesting that the violent act was responsible for renewed well-being (p. 41).

Just as “all the many moments and manifestations of Italian cinema are manifestations, as well, of a complex sociological reality” (Burke, 2017, p. 6) so significant social movements such as feminism and legal reforms to increase women's rights and protection under the law mean that Italian cinema today has moved away from light comedic representations to more politically engaged narratives that foreground instead the pervasive nature of domestic violence that remains deeply rooted in long-term acceptance of such behavior not withstanding cultural and legal changes.^4^ Unlike the filmmakers of the late 1950s to 1970s who did not “intend to denounce the behaviour they represented” (Patriarca, 2010, p. 203) in their violent male characters, the filmmakers of the new millennium discussed in this article engage in a more overtly political statement. Not only do they move away from narratives whose plots centered on the concerns and predicaments of Italian masculinity which “even when their predicaments involve relations with femininity it is the masculine viewpoint that prevails” (Gundle, 2017, p. 207), but they also challenge prevailing cultural notions. The three male directors, working closely alongside their female collaborators—women authors, screenwriters, and producers—place the female's story and her gaze at the center of the film. Furthermore, the visual, audio, and editing strategies employed in these films encourage a form of sensory and affective viewing, which brings us closer to the protagonists’ experiences. It is through various forms of viewer alignment or engagement that these narratives, foregrounding unacceptable practices of intimate partner violence, become exemplary narratives that can empower women both on-screen and off-screen.

In the unravelling of their narratives, the films ultimately not only denounce the violence but challenge the notion of domestic violence as a fixed and unchangeable condition for women. While the films bring to light the deeply seated cultural and political realities that obstruct female empowerment and dignity and the challenges and fears of women who try to reclaim their lives, what each film highlights is the possibility of *la vita possibile,* an afterlife to a past life of violence, and one that comes about not through grand-scale social transformation but through various forms of female solidarity.

## Relationality, Care, and Resistance

The films discussed below draw attention to how solidarity and relationality, theorized in a range of feminist discourses; empathy between women (both within the filmic narrative and beyond); and an accompanying ethics of care become instrumental in combating the isolation, guilt, and associated emotions that afflict women in emerging from violent relationships. In Italy, relationality is theorized in the Italian feminist practice of *affidamento* (entrustment), which grew out of the Italian women's movement of the 1980s. Mainly conceived as a socio-political practice (Giorgio, 2002) designed to enable women in the public sphere, it recognizes the sense of agency and empowerment that can emerge from vertical relationships (based on a maternal model) between women, often different in age, class, or even ethnicity. A woman with authority, knowledge, or skill can be in a position to empower the other woman who entrusts herself to her. These power dynamics are not unilateral, and in different circumstances, the relationship between the one who entrusts and the entrusted may well be reversed (Cicioni, 1989). Judith Jordan (1991) in her work on women's psychological development focuses on the mutuality of such relationships and describes the fluidity of these mutually beneficial relationships: one woman is both affecting the other and being affected by the other; one reaches out to the other and is also receptive to the other. There is an openness to influence, emotional availability, and a constantly changing pattern of responding to and affecting the other's state (p. 82). Akin to the notion of relationality and dependent on it is the ethics of caring. As Maria Puig de la Bellacasa (2012) argues, care is a practice, an enactment, a doing based upon creating a relation. It is an affectively charged and selective mode of attention that directs action. In *Caring,* Nel Noddings (1984) articulates an approach to ethics rooted in relationality, receptivity, and responsiveness, “Caring is largely reactive and responsive. Perhaps it is even better characterized as receptive. […] Whatever the [one-caring] does for the cared-for is embedded in a relationship that reveals itself as engrossment and in an attitude that warms and comforts the cared-for” (p. 19). According to Noddings, the person providing care reaches out to the one she offers care to, moving away from herself, “When I receive the other I am totally with the other. […] The other ‘fills the firmament’” (Noddings, 1984, p. 32). The relationship, however, is reciprocal, because the one cared for completes the relationship by accepting the care that is offered. In Carol Gilligan's (1992) seminal text *In a Different Voice*, care is configured as a female, relational approach to ethics where attachment and compassion take precedence over impartiality and rights that define a justice-based approach. Within the narratives of the films discussed below, the notion of relationality and of care, operating outside what is configured as an ineffectual public justice system, is based on a female character reaching out to another who is open to receive their care, advice, and support and becomes fundamental to their resistance to violence.

## Ferzan Ozpetek's *Un Giorno Perfetto:* Female Solidarity Amid a Massacre

Ferzan Ozpetek's film is an adaptation of Melania Mazzucco's (2005) book of the same title, written prior to the period of widespread media attention to gender-based violence in Italy. Ozpetek, a very well-known director in Italy, was approached by the influential producer, Domenico Procacci, to adapt Mazzucco's novel into a film. As Stefania Lucamante (2010) suggests, Mazzucco's work joins other works by contemporary women writers, “whose investigation of violence takes the form of a distinctive authorial voice that denounces violation of women. Underreported aspects of women's lives within their own families beg for analysis and discussion rather than silence and rhetorical images” (p. 400).

In his adaptation, Ozpetek retains the novel's choral nature intersecting a number of characters and stories with institutional Rome positioned as the backdrop of patriarchal power and politics that together seem to conspire to oppress women. Ozpetek's deliberate choice of adding more female characters to those present in the novel contributes to the empowering impact of female solidarity within the narrative itself and is complemented by an affective visual style, which invites the spectator to align themselves with the female protagonists. The narrative takes place in the course of a (less than perfect) day as the title suggests, and is told retrospectively, following the generic practice of a certain kind of crime film which begins with the aftermath of a crime and jumps back to the events leading up to it. From the film's opening scene, the viewer is drawn into what promises to culminate in a horrible event, as the police arrive at the door of Antonio's apartment, following a neighbor's report of screaming and gunshots. The action then backtracks to 24 h earlier. Ozpetek introduces, in their various contexts, the many characters of the narrative including the two main protagonists: Emma, played by Isabella Ferrari, the estranged wife of Antonio, played by Valerio Mastandrea. We learn that a year earlier Emma had taken her two children and left Antonio and their home, the site of repeated acts of domestic violence. The opening of the film illustrates the entrapment and oppression that Emma continues to experience. Her framing, through the glass window of her mother's apartment exposed to Antonio's stalking gaze in the street below, accentuates her captivity and continued vulnerability to his violence. The film clearly highlights that leaving an abusive relationship does not guarantee an end to the threats posed by intimate partner violence. The court orders meant to protect Emma seem useless as Antonio considers himself above the law and retains the authority bestowed upon him by a well-entrenched patriarchal tradition, represented in the film by the old political guard that surround him: his politician boss, his former police department friends, and the men who question Emma's denunciation of her husband. Emma is repeatedly preyed upon by a tormented Antonio, who intrudes into every aspect of her life. He is one dimensionally configured by Ozpetek as the typical male abuser, unable to accept the disempowerment that results from his wife having finally found the courage to leave him. He calls her repeatedly at her place of work and at home where he shouts insults at her mother when she answers the phone. On the fated day, he waits for her outside her office building where she reluctantly agrees to talk to him. Inside his car, he pleads for reunification, his claims of reformed behavior clearly untrue as despite her protests to stop the car, he drives to the banks of the Tiber River and drags her into the bushes. The camera assumes a shaky, handheld style as it follows the couple into the reeds staying so close to the bodies that their parts become indistinguishable from the whole. The spectator is denied what could have easily been a voyeuristic scene, left only to hear the sounds that reveal Emma's attempts at resistance and escape. According to Scott and Van de Peer (2016) in their study on female solidarity in Francophone cinema, this type of aesthetic that includes the use of close-ups and ambient sound, aligns the spectator with the woman on screen. The “affective alignment or proximity of the bodily experience of other woman” transmits a sympathetic “feeling-with,” which encourages a sympathetic bond between the protagonist and the spectator (p. 174). Emerging from the bushes into full sunlight, Emma manages to escape Antonio. A long shot of her running away and up the staircase that ends in a low angle shot framing her visually and symbolically towering above Antonio, collapsed at the side of walkway, reverses the power dynamic and strips him of any power and authority over her. Spectator sympathy remains with Emma as she cleans her bloodied lip and tries to conceal the visible scar of this invisible violence with make-up. As Emma's reflection in the bathroom mirror stares back at us, holding our gaze, the spectator's alignment and sympathy with Emma are reinforced.

Having lost authority over his wife, Antonio assumes instead self-delegated paternal authority over his children*.* The unsanctioned and unquestioned unification of father and children at the birthday party prefigures the tragic outcome signaled from the beginning of the film and reaffirmed by Antonio's repeated explosions of violence. As the children watch *The March of the Penguins* with its explicit references to parental obligations and protection, the violence alluded to begins to unfold in the family home. Antonio stares straight into the camera, gun in mouth, before the shooting begins. As earlier in the film, the viewer is denied the sensationalized, voyeuristic view of the violence as it unfolds; we hear the shots but do not witness the shooting. It is only later that the spectator, positioned behind the camera that follows the police into the house, incrementally pieces together the extent of the violence as the camera slowly pans the bloodied sheets, Antonio's hand holding the gun he used to shoot himself, the blood-stained clothing of the small lifeless body of Kevin, and finally a shot of the body of Valentina still breathing being taken away in an ambulance.

While the narrative structure and selection of main actors (both Mastandrea and Ferrari are popular among Italian audiences) direct the viewer's attention to the main thread of the film outlined above, the intersecting secondary narratives and supporting characters are what prevent the film from being simply “un pugno nello stomaco” [a punch in the gut], as many critics described it when it was first screened at the 2008 Venice Film Festival, and provide instead a redemptive final message. Complementing the main narrative, which illustrates Antonio's downward spiral where the ultimate perversion rather than preservation of his role as husband, father, and public figure of authority leads to tragedy, are the additional subplots that connect the female characters of the film through a strategy of entrustment and self-preservation that sits in opposition to the betrayal and lack of support Emma experiences with the very institutions that are meant to protect her: marriage and the legal system. Two secondary characters, Mara (adapted from a male character in Mazzucco's novel) and Maja, are positioned in relation to Emma and foreground the centrality of female solidarity to the film.

Emma's fortuitous encounter with Mara*,* her daughter's teacher, occurs as she is walking through Rome's upscale shopping district following her violent encounter with Antonio. Emma and Mara smile at each other through a shop window, and the framing of the shot-reverse-shot sequence sets them up as mirror images of each other. As they greet one another outside the shop, the two-shot further establishes their affinity through the chromatic paralleling of Mara's red scarf and Emma's red blouse. This paralleling also signals their difference, with Mara's reserved nature suggested by her closely wrapped scarf, and Emma's sensuality by her loosely fitted silk blouse and matching bright red lipstick. The narrative establishes an immediate bond between these two women of decidedly different classes and backgrounds, who become sources of mutual support for each other on this less than perfect day for both of them. Their initial dialogue, based on the two women's shared concern for Emma's daughter, Valentina, evolves into a conversation in which Mara listens attentively to Emma's many self-doubts that are a consequence of her broken marriage and societal expectations and later, to the history of her relationship with Antonio. Emma's account of her roller-coaster life and misfortunes reveals not weakness and failure as she suggests, but the strength and passion that position her to face the challenges that await. A dejected Mara, whose lover has just ended their relationship over the phone, accompanies Emma home and then across Rome in search of her children. The Mara-Emma encounter transforms into an intimate journey, both in and out of time, a hiatus dominated by female gestures and words that intercuts with other episodes leading up to the ending of the film. When the two women part, each thanking the other for the mutual support provided in the course of the evening, they each utter the other's name, affirming their agency against the backdrop of an unforgiving patriarchal Rome. Left on her own, Emma re-appropriates the nocturnal urban space, walking through the street with a renewed sense of autonomy and freedom, accompanied by the sensory delight of eating an ice cream, suggesting the renewed pleasure of consuming rather than being consumed.

If the Mara–Emma fortuitous encounter is the example of female solidarity in the film that essentially “saves” both Emma and Mara, a parallel narrative, that of Maja, second wife of the politician Fioravanti, places Emma in the position of a savior of sorts. Parallels are drawn between Emma and Maja despite their difference in class and in physical appearance. Maja is entrapped in a tower of her own, in what comes across as a cold, loveless, and abusive marriage where she plays the role of a trophy wife. Her physical and psychological isolation is suggested by shots which frame her within the walls of her palatial home in one of Rome's most exclusive communities, dressed in tasteful, elegant clothing or in the monumental buildings which represent the institutional power held by her husband. Maja's body is frail and contained, representing what Bakhtin (1984) defines as the classical body: an image of a closed individuality that does not merge with other bodies and with the world. Emma's body on the other hand corresponds to Bakhtin's “grotesque body that outgrows itself, transgresses its own limits. … [It] is not separated from the world by clearly defined boundaries” (Bakhtin, 1984, pp. 26–27). Emma moves with ease around the spaces of the city, from her home in the margins of the city, to the working-class neighborhood of Tiburtino, to the city center. Her “inappropriate” clothing, her short skirts and tight tops (which throughout the film attract disapproving gazes and comments), is a sign of Emma's transgressive nature and a vitality which leads to a re-appropriation of agency. While on the one hand Maja voices her disapproval of Emma's appearance to her husband, close-ups that capture her gaze on Emma tell a different narrative, one that suggests intrigue and admiration as she recognizes in Emma a grounded strength that she has lost since her marriage to the paternal and paternalistic figure of Fioravanti.

The oppressive behavior displayed by Antonio and Fioravanti exemplifies the abuse of power and the pursuit of control based on entrenched social mores that transcend class. Theirs is a distinctly male expression of violence, domination, and entitlement that they seem to have passed down to the next generation of males, the bullying classmates of Emma's son Kevin. Emma, Mara, and Maja counter this violence by forming unexpected alliances that provide them with the possibility of imagining a way of reclaiming their bodies and their lives. Emma and Valentina survive Antonio's rage, while a pregnant Maja, brought face to face with a portrait of herself pre-marriage, contemplates leaving her husband. Mazzucco's narrative voice that comes through in the film envisages what Lucamante (2010) calls “the ethical duty on the part of women and male authors and filmmakers to expose the violence of all kinds still oppressing and repressing Roman women and Roman society” (p. 400). Ozpetek's *Un giorno perfetto* exposes that violence, but ultimately turns its attention to strategies for overcoming it. Emma, Maja, and Mara, directly or obliquely, empower each other through strengths that transcend their social or class position, precisely because their relationships supersede failed institutional structures, cultural stereotyping, and stifling societal mores.

## Andrès Arce Maldonado's *Dentro*: An Ethics of Care

*Dentro,* a small production film, directed by Andrès Arce Maldonado and written and produced by Sibilla Barbieri, was first screened at the Roma Tre Film Festival in May 2017. The film confronts the issues of domestic violence through the intertwining stories of two women: Ada, a wealthy upper-class woman, and Liliana, her housekeeper. The title of the film is significant, for aside from the scenes that follow Liliana on her meandering through the city of Rome, the entire film is shot inside, behind the many locked doors that conceal acts of violence or windows that highlight barriers to accessing the outside world. Both of the protagonists’ claustrophobic domestic spaces are cages that imprison them, spaces where they are enslaved, preyed upon, and where they become victims of invisible violence. Liliana's home is modest, cluttered and dark. High angle shots of her lying in bed entrapped within her husband's embrace or locked in the bathroom to avoid him, showcase the home as a prison from which it is impossible to escape her husband's rage. Ada on the other hand is kept like a bird in a golden cage inside her elegant upper-class abode where she is subjected to her husband's violent desires. Her movements are carefully monitored, even her exercising takes place on a treadmill inside the house where her husband controls the settings on the machine.

An alliance forms between these two women, different in age and class, as they come to recognize they are both entrapped in violent marriages in which they are controlled by the possession, fear, and a perverted form of “love” that their husbands impart. Like *Un giorno perfetto*, *Dentro* is cyclical in structure, ending in a slightly extended version of its opening scene where the identity of the unnamed victim of the news broadcast becomes clear. The first half of the film recounts Liliana's story from her perspective while the second part of the film tells Ada's story from her point of view. Where the two narratives intersect there is narrative accrual, that is while bits of narrative are repeated, a different perspective or extended episode clarifies an incomplete or unclear piece of the story.

The film begins steeped in Liliana's world, the camera locating us inside a darkly lit cramped kitchen where a clearly anxious Liliana is having her morning cup of coffee before crossing town to an upper-class house where she works. The narrative progresses alternating Liliana's daily work routine with the physical abuse she suffers on a regular basis when she returns home to a husband just released from prison. The beatings take place in cramped, dark interiors with the action itself intensified in the deliberately under-lit scenes. The violence is hidden from the viewer. As in the attempted rape scene in *Un giorno perfetto*, it is not through visual clues but rather through aural ones—the screams of the abusive husband, the cries of the victimized wife, the crashing of objects on the floor—that the viewer experiences the events, creating thus an affective experience which aligns the viewer with Liliana's experience. It is only after the violent events, through Liliana's reflection in the two bathroom mirrors, that the viewer gains visual access to the aftermath of the violence, the blood, the cuts, and the bruises on Liliana's face.

While the filming strategies keep the violence visually distant, they foreground instead the solidarity that is formed between Liliana and Ada and an ensuing ethics of care. When Liliana presents herself at work at Ada's home the morning following her husband's violent attack, Ada encounters the same bruised image of Liliana's face that the viewer had seen in the previous double-mirrored scene. As a result, there is a second alignment between the viewer and Ada, as the viewer participates in Ada's caregiving response, as she gently cleans the wounds and carefully applies make-up reinstating to Liliana a dignified image of herself. The mirror reflects back a two-shot image of Liliana and Ada gazing into the mirror and smiling at each other, thus sealing the bond between the one who administered and the one who received care.

The following night, a repeat incident, more severe than the first, silences Liliana leaving her immobile and given up for dead by both husband and viewer. However, Liliana survives, retrieved by a homeless woman from the bin where her husband had dumped her body. The near death experience leaves her understandably shaken and unequipped to trust the medical staff at the health center who encourage her to report her husband. Traumatized and afraid, she does not return to the site of abuse but wanders aimlessly through the transient areas of Rome, from the underpasses to the train station to the Tiber River, joining the ranks of the other transients and homeless, temporarily avoiding the impending violence. The film deliberately plays on the viewers’ assumptions of the location of danger. Defying the popular belief that danger lurks in the shadows and is propagated by marginalized individuals, the film associates small acts of kindness, such as the offering of food and shelter, with a homeless man, as it proposes that the monster is not outside, but rather inside, as the title of the film suggests. Liliana's prolonged roaming eventually leads her back to Ada's home where she voices her deep-seated will to survive. Once again identifying with Liliana's plight, Ada continues in her role as caregiver. Her privileged socio-economic position means she can enable Liliana's escape from Rome. She offers her all the cash she has in the house as well as her wedding ring ensuring that Liliana will have the financial means to reach the safety of her daughter's home in Paris. From the terrace of her luxury apartment, where she remains imprisoned, Ada watches as Liliana sets out on her way to a new *vita possibile*.

At this point the narrative rewinds, with the initial encounter between Ada and Liliana being replayed, this time from Ada's point of view. The intangible sense of something not quite right in Ada's fairy-tale marriage slowly unravels through the scenes of bondage and physical mauling that define Ada's sexual relations with her husband, and where Ada is made to blame for Claudio's sexual impotence. Left alone to care for her wounds, the camera moves very close to Ada's scarred back, almost touching it, as Ada's own hand reaches back to touch the scars thus engaging the viewer in a similar, haptic experience, allowing us to participate first in feeling the pain and then in the administering of care. As Lant (1995) asserts, cinema is haptic both because of the camera's touching or penetration of the world, like the surgeon's internal handling of the body, and because of film's physical impact on the viewer. As we participate in this haptic viewing, we engage in an embodied, emotional experience.

As indicated above, there is a cyclical pattern to the film. As the narrative advances and we witness repeated scenes of sado-masochistic sexuality, parallels between Ada and Liliana become clearer, as does the meaning of Ada's response to Liliana's statement when she appears on her doorstep after her urban meandering: Liliana: “I don’t want to die”; Ada: “Neither do I.” The final scene brings us back to the beginning in an extended episode that explains the opening in which a news broadcast announces the discovery of the body of an unidentified woman in her 50s. This time, instead of simply hearing the announcement in the distance, as in the opening scene, we view the broadcast on the television screen along with Ada. With her, we see the familiar blanket that Liliana had wrapped around herself as she roamed through the city. Melodrama gives way to horror as a still camera captures Ada running, knife in hand, from the kitchen onto the terrace; her husband following her; then they run back into the house and into the camera's view. A struggle partially hidden from view ensues and leads to a high angle shot of the two bodies collapsed on the living room floor with blood oozing through Claudio's shirt. In contrast to the scenes of care, the viewer is kept at a distance from this rapidly developing drama. Ada's unexpected outburst and action, like other unexpected revelations (the scene of Antonio desperately crying, thinking he has killed Liliana) is not analyzed, condemned, or condoned—it is simply represented as raw emotion.

In its construction, the film parallels the violence in these two women's lives, and it emphasizes not only the consolation but also the strength that each provides to the other. Scars and bruises are shared and tended to, either by the other or by the victim herself, who sees the inscription of the violence inflicted on her body reflected back to her in a mirror. Liliana allows herself to trust Ada when she can trust no one else, to let Ada care for her physically, emotionally, and financially, and in return for that trust Ada gains an understanding of her own dilemma and attempts to ensure Liliana's safety. However, in a society as ruthless and merciless as that of *Un giorno perfetto*, Ada can facilitate but not guarantee Liliana's survival. Liliana, though finally committed to survive at all costs, found the resolve to leave her husband, though ultimately, she was unable to fight back against whoever it was who finally deprived her of *la vita possibile*. In a final act of revenge for Liliana's death, and in her own desperate attempt to live, Ada lashes out on her jailer and abusive husband. Opting for an ending that confronts violence with violence, Berbier (2017), the film's producer, explains at her presentation of the film at the Roma 3 Film Festival:

Women all too often marry their abusers, but rarely do they denounce them. We realize that the topic is extremely delicate, and in no way do we want to either devalue the pain of the victim or support acts of violence. But at the same time, there is something lacking in the debate [around social violence] and this moved us to make a film that confronts a dark side that is difficult to talk about.

## Ivano De Matteo's *La Vita Possibile*: The Journey to a New Life

As with the two films previously discussed, Ivano De Matteo's film, *La vita possibile,* is a powerful denunciation of domestic violence and the society that breeds it. While also drawing attention to the impact of domestic violence on children, the film is above all an affirmation of the role female solidarity plays in paving the way to a new life. The director's third feature film, written by his partner Valentina Ferlan, provides a new angle on his exploration of dysfunctional family dynamics undertaken in his previous films *I nostri ragazzi* (*The Dinner)* (2014) and *Gli equilibristi* (*Balancing Act*) (2012). As in Ozpetek's case, the director's reputation in Italy and the success of his previous films along with the casting of two of Italy's most famous female actors, Margherita Buy (Anna) and Valeria Golino (Carla), in the title roles ensured its wide theatrical distribution.

As the title of *La vita possibile* suggests, the film was made with the deliberate intent of illustrating a way out of the psychological and physical vortex of domestic violence. In interviews, De Matteo has repeatedly declared that his decision to base the film on the aftermath of leaving a violent relationship was inspired by both the actual account that motivated the film and by the many accounts provided by women he spoke to in the preparation of the film (Coming Soon, 2016). Most did not want to talk about the violence itself, but rather wanted to focus on the difficulties and challenges encountered when left on their own to face the aftermath.

Hence, the film begins with the only explicit scene of domestic violence, witnessed by Valerio, the victim's son, which provides the catalyst for Anna's decision to leave Rome. In the opening scene, the camera follows Valerio in an extended long shot, initiating from his point of view, that frames his father and mother in the background, as the father prepares to punch her in the stomach. As she crumbles to the floor, a shot-reverse-shot sequence alternates close-ups of Anna and her son, as she looks at Valerio and calls out his name before the camera turns to Valerio and pans down his boyish body, a pan that reveals his emotional distress evidenced by his wet track pants. From this powerful scene, we cut to a shot of mother and son seated on a train, initially facing backwards, representing the process of leaving their past life behind, and then forward facing, en route to a geographically distant destination and to what the title suggests: a possible new life. This new life is facilitated by the generosity of Anna's long-time friend, Carla, who welcomes them in her small Torino apartment, offering psychological support to her friend and forging a bond with Valerio. The slow-paced film tracks the challenges that mother and son face as they try to start their life anew: the practical challenges of Anna finding a job, of Valerio making friends and adapting to a new school, along with the emotional challenges, of trauma and loss, of accepting the end of a relationship and recovering from the abuse that persists in the memories and dreams that haunt them both.

The character of Carla, the free-spirited, long-time friend who provides warmth, empathy and material support, is posited as instrumental to Anna's survival. Since the film is narrated in a linear fashion, Carla's role has an additional fundamental narrative function as her exchanges with Anna fill gaps in the viewer's knowledge about the repeated incidents of violence Anna suffered and her husband's refusal to respect the court order to keep his distance. The trusted relationship also lends itself to frank conversations which reveal the many vacillating feelings based on competing emotions that victims of intimate partner violence suffer. Carla is the force that prevents Anna from returning to the site of abuse in moments of self-doubt and guilt. It is Carla who reminds her, and educates the viewer, of the dangers of returning to her husband, making clear references to the images conjured up in sensationalized media stories that Anna and a viewing public are all too familiar with: if she returns she will end up in a plastic shopping bag chopped up into bits and pieces. It is the friendship and space that Carla provides that assist Anna in overcoming ongoing obstacles including battling the senseless bureaucracy of the institutions meant to protect her and her son that instead require paternal consent. Dialogues with other secondary characters also provide insight into the past, and to the trauma and mixed emotions experienced by Anna's son Valerio who vacillates between longing for his father and recognizing the impossibility of return. Valerio forms a bond with the former foreign football player turned tavern owner who befriends him and eventually assumes the semblance of a surrogate father figure. Mathieu is for Valerio what Carla is for Anna, a trusted figure to whom he can reveal elements of his traumatic past. He is able to articulate to Mathieu, and to the viewer, what he had kept to himself: that they fled because his father would beat his mother often, once smashing her head onto a table.

The film juxtaposes dialogues and confessions with long gaps of silence and reflection that mirror the processes undergone by the characters in the film. *La vita possibile* is about the long and slow process of working through trauma, in this case the trauma that haunts both Anna and Valerio. To render this, the film divides its attention between the two characters, often alternating images of Anna and Valerio, as they walk or ride through the streets of Torino, with long shots that include the city's monumental buildings in the background as a reminder of institutional power or the unpopulated banks of the river that highlight their isolation. The slow pace of the film, the melancholic tone, the desaturated images, and the autumnal season reflect their tenuous in-between position. Anna wears an indelible mark of the violence she was subjected to on her face, the scar on her forehead that we first see in the mirror on the train, but additional psychologically penetrating close-ups throughout the film reveal a haunted expression that suggests the memory of her trauma. In *Beyond the pleasure principle*, Freud (1975) contends that trauma is an experience that repeats itself, that is not located in the original violent event, but in nightmares and repetitive actions of the survivor; trauma is a wound of the body and of the mind which can be dramatic and even life threatening. Valerio's own trauma is signaled by his nightmares and anguished screaming in the night and his withdrawal from his mother.

Having physically escaped Rome does not guarantee freedom from violence. The film records the omnipresence of male violence in the public as well as the private space. An early reminder of this comes in one of the first scenes of the film relayed through Valerio's point of view; from the balcony of the bedroom in his new secure domestic space, Valerio witnesses a violent altercation between owner and customer emanating from the bar below. Later, the omnipresent danger faced by women in the nocturnal urban space is highlighted in a scene in which Anna waits for the bus to take her home from her night job. She finds herself situated between two potential incidents of violence. The camera frames her literally between an unruly gang of drunk young men lurking meters away and a car that pulls up beside her and driven by one of Carla's friends, who had made advances on her at a dinner party. Anna is trapped as the camera captures her gaze fluctuating between the various predators. As she accepts a ride home with Carla's friend, she is clearly choosing between the lesser of two evils, the likelihood of having to respond to an unwanted sexual advance by the man she knows over the potential unknown violence of the threatening gang.

Valerio is also exposed to the pervasive presence of nocturnal masculine violence. The only figures that populate the park he traverses regularly are two young Eastern European prostitutes, one of whom befriends him and whom the young Valerio is smitten by. One night he follows her after she is picked up by a client. The camera captures Valerio's horrified expressions as he watches the sexual encounter before he takes off at full speed on his bicycle into the night. The image of the young prostitute waving him away mirrors the male violence of the opening scene in the film and highlights his frustration over his inability to protect the women he loves from predatory male behavior.

As the narrative progresses, the film turns its attention from Anna to Valerio as he begins to find a place for himself in the company of his peers, the neighborhood boys*.* An increasingly settled environment allows Anna and Valerio to acquire the beginnings of a renewed emotional and financial independence as they make plans to move out of Carla's protective space into a place of their own. The film's final scene signals a possible alternative future as Anna looks out the window to the street below as a content Valerio runs off with his new friends, having been reinstated into an alternative stable life outside the walls of the violent traditional patriarchal family left behind. While on the one hand the ending focuses on a rosy and optimistic event, the fact that Anna and Valerio will continue to be haunted by the specter of what has happened is signaled by a shot earlier in the film at Anna's place of work. In a serene scene suggesting the acquisition of a new familial balance, as mother and son go about their daily chores, she is cleaning windows and he is doing his homework, we note a huge mural on the wall featuring a dominating devil-like figure, lurking in the background, distant but omnipresent.

## A Cinematic Space for Reflection and Resistance

The three films discussed in this article create a space for reflection and contemplation not just on the pervasiveness of intimate partner violence in Italian society, but, more importantly, on the possibilities of resisting and responding to it. As such, they contribute to a new sub-category of Italy's revived *cinema d’impegno,* or politically engaged cinema. Many filmmakers in Italy today are addressing power dynamics between the politically empowered and the underdog, as they manifest themselves in both the public and the private space and between dominant and subaltern groups. Italian political cinema no longer consists primarily of films that reinterpret explicitly political and public male figures, but includes stories of disempowered or socially marginalized individuals. It is here we locate a space for films that address various forms of violence against women. Films like Francesca Comencini's (2004) *Mobbing* and Marco Tullio Giordana's (2018) *Nome di donna* (*Woman's name*) as denunciations of workplace abuse, adhere to a certain type of female-centered film, where the female protagonist resists against all odds and achieves legal recompense and recognition through the public justice system. The three female-centered films discussed in this article propose a different, less heroic-driven narrative structure, highlighting instead the importance of solidarity among women as an instrument to combat the isolation, guilt, and associated emotions that all too often prevent women from seeking or finding *la vita possibile*. Past and present Italian capitals, seats of political power and institutions that fail to address women's practical or emotional needs form the backdrops of the films, and intimate partner violence comprises the shared context and experience of the films’ protagonists. However, unlike many melodramas or horror films that “are often understood as packaging female pain and suffering as ‘spectacles’ to be consumed by voyeuristic spectators” (Scott & Van de Peer, 2016, p. 170), the films deliberately distance themselves from sensational representations of violence. As we have seen, the films adopt a range of aesthetic choices that include close-ups, two-shots, and following shots that encourage “a sense of physical and emotional proximity” (Scott & Van de Peer, 2016, p. 191) and “imagined engagement or identification” (Scott & Van de Peer, 2016, p. 172). Alongside character and narrative development the films provide a tactile and sensory viewing experience. Women on screen are provided with a space for dialogue and physical embraces, faces and bodies are reflected in mirrors, and their reflections stare back diegetically at them and extra-diegetically at us, holding our gaze with an intensity that speaks to women beyond the narrative to defy the violence they have experienced. Their bodies, looked at, touched and caressed by the camera are not only sites of repeated violence and injury, but also sites of care and recovery. The care offered and accepted and the make-up applied by a caring Ada to Liliana, or by Emma or Anna, indicate women's desire to heal, to reclaim their bodies, their mobility, and their agency. As Goldberg (2001) affirms, “If they are to be politically effective then cultural representation of pain and terror must achieve a fragile balance between the difficulty of presenting that which is too painful to be heard—and the imperative of presenting that very material in the service of […] resisting continued enactment of such violences” (p. 246). The films discussed attempt to achieve this very balance and create a space for rendering visible the physical and psychological scars.

## Conclusion: Turning Italy Around

Turning to the title of the film with which I began my discussion on domestic violence in Italian cinema, the fiction films examined in this article, like the documentary *Violenza invisibile*, reinforce the imperative that “gli italiani si voltano,” that is Italians “turn around” in both senses of the word. Italy must turn around a culture where gender*-*based violence is deeply rooted in the homes of the privileged and the underprivileged and the stalkers and abusers must recognize and seek therapy for their behaviors; they must turn their gaze not on women as objects of desire and possessions, but on themselves so that we can emerge from what De Bonis (2018) has called “this black hole of dependence, of automatism fixed in the collective memory. To exit from this tunnel is a challenge for civilization, for humanity.” As the films suggest, while the support for victims of violence has increased significantly, the patriarchal legal systems and attitudes that still protect abusers and the lack of attention to the much needed rehabilitation of men in Italy compared with other countries is sobering. As Marina Valcarenghi, a Milanese psychiatrist argues in her book *Ho paura di me* (2009), succeeding in setting out a serious strategy with violent males, stalkers, rapists and pedophiles is not easy. In addition to the lack of adequate facilities and the repression of these forms of behavior, social stigma prevents many therapists and psychologists from taking on these patients. Valcarenghi and other psychologists suggest that in order to “cure*”* and “prevent*”* this ongoing culture of violence, Italian society must first understand that such violence is not attributable primarily to deviant behavior but to behavior which has been normalized.

As Italians work collectively toward recovery and grand-scale social transformation that will remove the haunting mural lurking in the background of De Matteo's film from the collective imaginary, we recognize the place that the arts play in this process. As Gabrielle Schwabb purports, the arts can be transformational in the sense that by endowing knowledge with a symbolic form of expression they not only change its status but make it indirectly accessible to others. Visual and performing arts continue to play a major role in raising awareness and stimulating public dialogue and debate around gender-based violence as have numerous media initiatives sponsored by the ministries of culture and equal opportunity.^5^ The red bench project, *Panchine rosse: contro la violenza sempre,* which since 2016 has seen the emergence of red park benches in parks and public spaces across Italy, is but one colorful symbolic example of a nation-wide initiative that has spread throughout Italy reaching city centers, urban peripheries, and smaller communities. Red benches, with their visual affiliation to blood and violence, have become a recognizable physical symbol inscribed on the Italian landscape, an omnipresent reminder of the absent or wounded woman on the bench, of the need to sit down and listen to these women, and ultimately a plea for widespread commitment to providing support to female victims of violence.

In addition to the many artistic initiatives that raise public awareness, television advertising campaigns have reached out to female victims to encourage them to take the first step to a violence free life. The ad campaign #Liberapuoi [You Can Be Free] encourages women to seek support through the national helpline (1522). The November 2019 30-s television ad featured a scene of an anxious mother crouched in a dark room with her daughter calling the helpline, and a subsequent scene of the same woman, smiling now, relocated with her daughter in a brightly lit apartment—new house, new job, new life. In March 2020, during the first national lock-down measures of the pandemic, the Italian government responded to the anticipated increase in violence with a special awareness campaign on public television channels urging women to make use of the toll-free helpline (1522) and its related chat and app. The ad featured nine well-known Italian entertainment personalities, seven women and two men, each delivering their message by looking straight into the camera and addressing female victims. The ad's message consisted of each speaker providing a fragment of the domestic violence picture. Beginning with the often cited statistic that every three days a woman dies of violence in Italy, it continued by stressing the problematic context: during lock-down everyone must stay home and follow the rules, while we all know that the home is not necessarily a safe-haven for women. More statistics on women who have died as a result of intimate partner violence are provided before the ad turns to the second person form of address: “You can say no, even during the current pandemic emergency, you are not alone, anti-violence centers are open to protect you and not let you lose hope. You can ask for help, pick up the phone or download the app. We are there, we are together, always; there's always a way out.” The ad ends with each personality emphatically repeating the tagline identified with the campaign: “libera puoi.”^6^ The importance of the ad is indisputable in reminding threatened women that help is available, and calls to the number reportedly increased as a result of the campaign. However, a more nuanced message on the complexity of the process toward a violence-free life needs to delivered as well. As *La vita possibile* highlights through Anna and Valerio's journey, the transformation from the dark violent corridors of the home to a joyous renewed life is not immediate and is dependent on a complex network of people and events. As Ferelli (2013) states in her study on common practices in support centers for women:

There is no quick fix, and while acknowledging violence and leaving an abusive situation are important first steps, the process toward rehabilitation is a long one and is contingent on a number of considerations and on relations based on networks and trust. Distancing oneself from the abusive partner and ascribing to the support systems constitute only the point of departure of a long and sometimes difficult path toward their integrity, self-confidence and autonomy. To not consider the importance of this process, and of the long time that this might take, signifies simplifying and not recognizing the effects of this violence and further penalizing women.” (80)^7^

The films discussed, with their emphasis on the reliance of victimized women on trusted relationships that grow through repeated acts of care, highlight the importance of the emotional support “that produces in the subject the knowledge of being accepted and appreciated, the practical support allows for the material resources required, and finally the cognitive support furnishes information regarding the situation that allows the individual to keep it controlled and to manage it” (Ferelli, 2013, p. 82). As fictional exemplary case studies, these films strive to be at best transformational or at the very least to provide a life-affirming strategy for the still very problematic present, a strategy that highlights the centrality of female solidarity and care and advocates for intersubjective compassion that is possible between subjects from widely disparate backgrounds and experiences, both on and off-screen. A strategy that leads to *la vita possibile*.

## Notes

1. See: https://www.istat.it/it/archivio/161716 for a summary of statistics on violence against women provided by Istat (the Italian National Institute of Statistics). The summary reveals that violence against women continues to be a widespread phenomenon. According to the statistics, 6,788,000 women have suffered some form of physical or sexual violence during their lifetime. The summary suggests that this is a transversal phenomenon in Italian society.

2. See Torriglia (2002, pp. 58–66) for a discussion of films from the post-war period and early 1950s that present female characters with aspirations for independence and roles beyond that of wife and mother; Luciano and Scarparo (2013, pp. 432–436) for a discussion on the emergence of female directors (Lina Wertmüller, Liliana Cavani, Soria Scandurra, Dacia Maraini, Giovanni Gagliardo, and the Feminist Film Collective) in the 1960s and 1970s whose films examined the power struggles between men and women and the relationship of women to history and societal institutions, and raised public awareness of women's socially and politically disadvantaged status.

3. Cullen identifies Luigi Comencini's *Bread, Love and Jealousy* (1954), Dino Risi's *Poor but Beautiful* (1957), Vittorio De Sica's *Marriage, Italian Style* (1964), Antonio Pietrangeli's *The Magnificent Cuckold* (1964), and Visconti's late neorealist drama classic *Rocco and his Brothers* (1960) as examples of films featuring jealousy as a theme.

4. See http://www.salute.gov.it/portale/donna/dettaglioContenutiDonna.jsp?lingua=italiano&id=4498&area=Salute%20donna&menu=societa for details on laws regarding violence against passed in Italy since 1996; in 1996, a law passed declaring violence against women as a crime against personal liberty (as opposed to against public morality); in 2001, restraining orders were set in place along with state economic support for women victims of violence; in 2009, increased penalties for offenders and stalkers were enforced.

5. Examples of such cultural manifestations: In Milan, in Piazza Scala, a theatrical performance, “Le sedie” [The Chairs], was staged by the theatre company of inmates and former inmates and provided testimonies by women victims of violence who as a result of denouncing their victimizer found a pathway to a new life. In Padova, an exhibit of seven famous works of art exalting female beauty were repurposed to show scarred faces, black eyes, bruised necks, swollen lips; the seven panels suggesting that every woman is a work of art to be respected and not maimed. Yet another art installation entitled “Il luogo più pericoloso” [The Most Dangerous Space], consisted of hundreds of plates designed and painted by two artists addressing the theme of gender-based violence. Why plates? As gender-based violence inhabits the home, the very space that should be the safest, most intimate, and most welcoming.

6. The full ad is available here: https://www.youtube.com/watch?v=p_llVUk8iz0 Translation of the script is my own.

7. Translations of all Ferelli's quotes are my own.
